# The Maternal Microbiome and Gestational Diabetes Mellitus: Cause and Effect

**DOI:** 10.3390/microorganisms11092217

**Published:** 2023-08-31

**Authors:** Stephanie Dias, Carmen Pheiffer, Sumaiya Adam

**Affiliations:** 1 Biomedical Research and Innovation Platform (BRIP), South African Medical Research Council, Tygerberg, Cape Town 7505, South Africa; stephanie.dias@mrc.ac.za (S.D.); carmen.pheiffer@mrc.ac.za (C.P.); 2 Centre for Cardio-Metabolic Research in Africa (CARMA), Division of Medical Physiology, Faculty of Health Sciences, Stellenbosch University, Tygerberg, Cape Town 7505, South Africa; 3 Department of Obstetrics and Gynaecology, School of Medicine, Faculty of Health Sciences, University of Pretoria, Pretoria 0028, South Africa; 4 Diabetes Research Centre, Faculty of Health Sciences, University of Pretoria, Pretoria 0028, South Africa

**Keywords:** gestational diabetes mellitus, maternal microbiome, pregnancy complications, adverse maternal outcomes, adverse neonatal outcomes

## Abstract

Gestational diabetes mellitus (GDM) is a growing public health concern that affects many pregnancies globally. The condition is associated with adverse maternal and neonatal outcomes including gestational hypertension, preeclampsia, placental abruption, preterm birth, stillbirth, and fetal growth restriction. In the long-term, mothers and children have an increased risk of developing metabolic diseases such as type 2 diabetes and cardiovascular disease. Accumulating evidence suggest that alterations in the maternal microbiome may play a role in the pathogenesis of GDM and adverse pregnancy outcomes. This review describes changes in the maternal microbiome during the physiological adaptations of pregnancy, GDM and adverse maternal and neonatal outcomes. Findings from this review highlight the importance of understanding the link between the maternal microbiome and GDM. Furthermore, new therapeutic approaches to prevent or better manage GDM are discussed. Further research and clinical trials are necessary to fully realize the therapeutic potential of the maternal microbiome and translate these findings into clinical practice.

## 1. Introduction

The prevalence of gestational diabetes mellitus (GDM), defined as glucose intolerance that develops during pregnancy [1,2], is on the rise globally. GDM affects approximately 14% of pregnancies worldwide, with rates varying according to the population investigated and diagnostic criteria employed [3]. The increase in GDM prevalence is exacerbated by rising rates of obesity, sedentary lifestyles, and advanced maternal age [4]. GDM has been associated with an increased risk of short- and long-term adverse health outcomes in both mothers and their offspring [5,6,7]. Short-term complications in mothers include caesarean section, placental abruption, gestational hypertension, preeclampsia, and increased susceptibility to infections. Offspring exposed to GDM have an increased risk of macrosomia, fetal growth restriction, preterm birth, and neonatal metabolic dysfunction. In the long-term both mothers and babies have an increased risk of developing metabolic diseases such as type 2 diabetes mellitus (T2DM) and cardiovascular disease [5,6,7,8]. Despite a well-established association between GDM and unfavorable maternal and neonatal outcomes [5,6,7], the mechanisms that underlie this link remain incompletely understood.

Dysbiosis of the microbiome has been associated with numerous metabolic diseases, including obesity [9], T2DM [10], and GDM [11]. The microbiome refers to the collective group of microorganisms inhabiting various parts of the body including the oral cavity, gastrointestinal tract, skin, lungs, vagina, and the placenta [12,13], and has been demonstrated to play an essential role in various biological processes, including metabolism and immunity. Although microorganisms exhibit commensalism or mutualistic relationships with their host [14], they can disrupt normal physiological processes when dysregulated [15]. During pregnancy, a woman’s normal microbiota composition undergoes several changes to accommodate the growing fetus [16].

The role of the microbiome in the pathogenesis of GDM and adverse maternal and neonatal outcomes has received increased attention [17,18,19]. However, most studies that have investigated the microbiome during GDM and adverse pregnancy outcomes are observational and have failed to establish a causal role for the microbiome in the pathophysiology of these conditions. This review describes changes in the maternal microbiome during the physiological adaptations of pregnancy, GDM and adverse maternal and neonatal outcomes. Firstly, the pathophysiology of GDM is described, followed by delineation of changes in the maternal microbiome during pregnancy, GDM, and adverse maternal and neonatal outcomes. Lastly, the therapeutic potential of the maternal microbiome in relation to GDM and adverse outcomes, challenges and limitations of current studies, and avenues for future research are highlighted.

## 2. The Pathogenesis of Gestational Diabetes Mellitus

**Figure 1:**
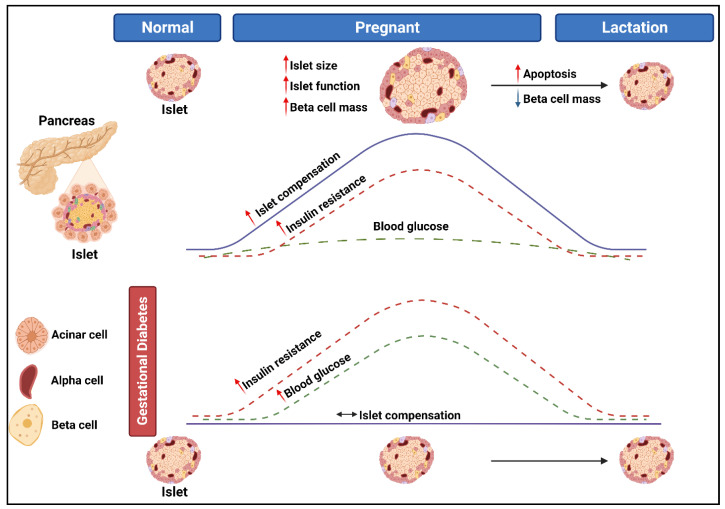
The pathogenesis of gestational diabetes mellitus (Created with BioRender.com).

GDM is a complex disorder, characterized by hyperglycemia, that is first diagnosed in the second and third trimester of pregnancy [20]. The pathophysiological mechanisms that underlie the development of GDM are not yet fully understood, although it has been proposed that GDM develops in women who are unable to adapt to insulin resistance [21]. During pregnancy, insulin sensitivity decreases, which is speculated to be an adaptive mechanism to meet the physiological demands of the developing fetus. The decrease in insulin sensitivity is mediated by the secretion of maternal and placental hormones such as human placental lactogen (hPL), estrogen, progesterone, placental growth hormone, cortisol, and prolactin [4]. To maintain normoglycemia, the pancreatic beta (β)-cells increases insulin production and secretion. However, in some women, the pancreatic β-cells are unable to compensate for the increasing insulin demand, leading to glucose intolerance and the development of GDM (Figure 1). Risk factors of GDM such as advanced maternal age (≥35 years), obesity, diets high in fats and sugar, ethnicity, hypertension, family history of GDM or T2DM, and personal history of GDM or polycystic ovarian syndrome, are associated with impaired β-cell function and/or insulin sensitivity [22,23]. Thus, it has been suggested that GDM represents a temporary unmasking of pre-existing metabolic disturbances.

## 3. Microbiome Alterations during Pregnancy

**Figure 2:**
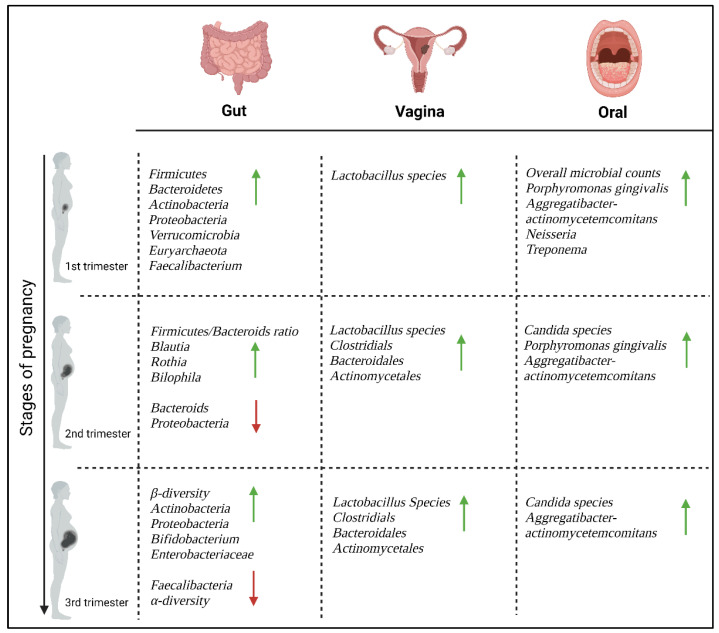
Maternal microbiome during healthy pregnancy (Created with BioRender.com). Green arrows indicate increased microbiota, red arrows indicate decreased microbiota.

Pregnancy is a complex physiological process whereby several metabolic, hormonal, and immune system changes occur to support the growth and development of the fetus and to prepare the mother for birth and lactation [24]. Coinciding with these pregnancy-related changes, the maternal microbiome has been shown to change dramatically at various body sites [16]. The majority of microbes reside in the gut and harbors hundreds of bacterial species, of which *Firmicutes* and *Bacteroidetes* are considered the most dominant bacterial phyla. In addition, the vagina and oral cavity provide important niches for distinct bacterial communities during pregnancy, which contribute to the immune system by defending against potential pathogens [16]. Hormonal shifts during pregnancy, such as elevated levels of progesterone and estrogen affect the pH levels of the vaginal environment and impacts the growth and diversity of the vaginal microbiota [25], while immune, metabolic, and other factors such as diet, lifestyle and hygiene are known to influence the gut and oral microbiome [26,27]. These changes in the maternal microbiome are vital to maintaining a healthy pregnancy and may also support fetal development and impact the metabolism, behavior, and immunity of the offspring [16]. However, these same essential changes may also make women more vulnerable to immunological and infectious diseases during pregnancy and the postpartum period [28]. In addition to the vaginal, oral and gut microbiota, the placenta plays an important role in the regulation of immunity during pregnancy. However, the existence of the placental microbiome has long been a topic of debate and controversy, and has been suggested to reflect the vaginal or skin microbiome [29]. Changes in the maternal microbiome are illustrated in Figure 2.

### 3.1. The Gut Microbiome

The gut microbiome is increasingly recognized as a significant contributor to metabolic health [30] and undergoes various alterations during pregnancy [17,31]. During the first trimester, the gut microbiome mirrors that of healthy, non-pregnant women, but shifts substantially in composition and structure over the course of pregnancy [32]. In healthy pregnant women the gut microbiome is dominated by two major bacterial phyla, *Firmicutes* and *Bacteroidetes*, followed by *Actinobacteria*, *Proteobacteria*, *Verrucomicrobia*, *Euryarchaeota* and *Faecalibacterium* [33,34]. As pregnancy progresses, there are dramatic changes in the gut microbiome from the first to the third trimester. The main changes are represented by a reduction in the *alpha* (*α*)*-diversity*, which refers to intra-individual diversity, and an increase in *beta* (*β*)*-diversity*, which refers to inter-individual diversity [35]. Koren et al. (2012) reported a greater abundance of pro-inflammatory *Actinobacteria* and *Proteobacteria* levels, while bacteria such as anti-inflammatory *Faecalibacterium* and butyrate-producing bacteria, which are usually depleted in patients with metabolic syndrome, were decreased from the first to third trimester of pregnancy [33]. These findings were coupled with insulin insensitivity, weight gain and elevated levels of cytokines, indicating the presence of inflammation. According to Koren et al. (2012), increased levels of *Proteobacteria*, commonly associated with inflammatory conditions, and elevated levels of *Enterobacteriaceae* were identified in the third trimester of pregnancy [33]. To elucidate the role of the gut microbiota during pregnancy, fecal matter from the first and third trimester was transplanted to female germ-free wild type mice. Findings from the study demonstrated that the presence of specific gut microbiota was sufficient to induce inflammation and reduce insulin sensitivity and excess weight gain, suggesting that these microbiota may actively contribute to changes in host immunology and promote metabolic dysregulation [33]. Another study demonstrated an increase in the *Firmicutes*/*Bacteroides* ratio, *Blautia*, *Rothia*, and *Bilophila* and a decrease in *Parabacteroides* from the first to the second trimester of pregnancy [32,35]. These bacterial species commonly dominate the healthy human gut, and when altered have been reported to be associated with metabolic syndrome, inflammatory bowel syndrome disease and obesity. In contrast there is evidence to suggest that the level of *Bifidobacterium* increases in the third trimester [16]. *Bifidobacterium* is a type of beneficial bacteria that is commonly found in the gut microbiome, and helps to promote digestion, supports immune function and prevent the growth of harmful bacteria [36]. The increase in *Bifidobacterium* abundance improves the host’s ability to extract energy from the diet and store it in adipose tissue [37]. These discoveries suggest that the gut microbiome significantly influences pregnancy and the interactions between the host and microbes can affect the host’s metabolism, potentially leading to either favorable or harmful outcomes during pregnancy.

### 3.2. The Vaginal Microbiome

The vaginal microbiome plays an essential role in reproductive health by protecting against microbial and viral infections. Apart from preventing bacterial and viral invasion [38], the vaginal microbiome has been postulated to play a vital role in the timing of parturition, hormone secretion, and seeding of the infant microbiome during birth [39]. Under normal non-pregnant conditions, the vaginal microbiome is dominated by *Lactobacillus* species, including *Lactobacillus crispatus*, *Lactobacillus gasseri*, *Lactobacillus iners*, and *Lactobacillus jensenii*. The presence of non-*lactobacillus* genera, such as *Prevotella*, *Dialister*, *Atopobium*, *Gardnerella*, *Bifidobacterium*, *Megasphaera*, *Peptoniphilus*, *Sneathia*, *Eggerthella*, *Aerococcus*, *Finegoldia*, and *Mobiluncus* have also been demonstrated [40]. Throughout pregnancy, the vaginal microbiome serves protective roles such as promoting an abundance of *Lactobacillus* species and enhancing stability in the microbiota composition over time. *Lactobacilli* produce lactic acid to maintain a low pH (<4.5) and metabolites to protect against infections [12,25,41]. The increase in *Lactobacilli* may be due to the rise in estrogen levels during pregnancy, which increases the thickness of the vaginal mucosa and deposition of glycogen for *Lactobacilli* to utilize [12]. Additionally, the vaginal microbiota during pregnancy are enriched for *Clostridials*, *Bacteroidales*, and *Actinomycetales*, which is distinct from non-pregnant vaginal microbiome signatures [12,40]. The consistent stability of the vaginal microbiota throughout pregnancy indicates the significant protective role the microbiome plays in the health of both the mother and fetus. Indeed, evidence suggests that low levels of *Lactobacilli* during pregnancy is associated with bacterial vaginosis, a risk factor for preterm birth [42]. An improved understanding of the changes that occur in the vaginal microbiome during pregnancy could pave the way for treatment strategies to prevent pregnancy complications and adverse birth outcomes.

### 3.3. The Oral Microbiome

The oral microbiome consists of a wide range of microorganisms, which play an important role in maintaining oral health during pregnancy. An imbalance in the oral microbiome, which can be affected by factors such as pH, anaerobic conditions, diet, hygiene, and hormone levels [43,44,45], can lead to periodontitis, gingivitis, or systemic diseases [46,47,48]. During the first trimester of pregnancy, the oral microbiome contains significantly higher total microbial counts compared to non-pregnant women [49]. Fujiwara et al. (2017) reported an increased abundance of *Porphyromonas gingivalis* and *Aggregatibacter actinomycetemcomitans* in the first and second trimesters of pregnancy [49], which has been shown to play a role in insulin resistance and poor glucose control [50,51]. Similar to these findings, Lin et al. (2018) reported an over representation of bacterial species, *Porphyromonas gingivalis*, as well as *Neisseria*, and *Treponema* in pregnant women compared to non-pregnant women [52], while Borgo et al. (2012) showed a correlation with the presence of *Aggregatibacter actinomycetemcomitans* in the second and third trimester [50]. These findings indicate that the physiological changes in pregnancy may promote the proliferation of certain bacterial species in the oral cavity. Fujiwara et al. (2017) reported an increase in *Candida* species during the second and third trimester of pregnancy when compared to non-pregnant women [49], although, conflicting findings have been reported [53]. While *Candida* species are typically present in small amounts without causing harm, certain conditions can lead to their overgrowth, resulting in fungal infections in the mouth known as oral candidiasis or thrush [54,55]. Despite limited understanding of the mechanisms responsible for pregnancy-related changes in the oral microbiome, research findings suggest that pregnancy influences the composition of the oral microbiome. These results provide the basis for further research investigating the causes and implications of pregnancy-associated oral dysbiosis.

### 3.4. The Placental Microbiome

The placenta is a critical regulator of the prenatal environment and plays an important role in maternal health and fetal development [56]. Historically, the placenta was believed to be a sterile organ, and any introduction of microbes into the placenta was associated with adverse pregnancy outcomes such as preterm birth and bacterial vaginosis [57]. Recently, Panzer et al. (2023) reanalyzed placental microbiome data of 15 studies, with conflicting results from term deliveries [29]. Of the studies included in their review, only 13 included more than one term caesarean delivered placenta for comparison, and only eight included sufficient background technical controls to remove likely contaminants, with two of those studies lacking sufficient data to differentiate between samples based on gestational age at delivery. Of these, five studies concluded that there was no evidence for a placental microbiome in uncomplicated term caesarean pregnancies [58,59,60,61,62]. These studies linked the presence of placental microbiota to contamination during DNA extraction, well-to-well contamination during sequencing preparation or the use of contaminated laboratory reagents and equipment [58,59,60]. In contrast, four studies reported that a placental microbiome does exist [63,64,65,66], albeit without sufficient technical control data to exclude DNA contamination. Panzer et al. (2023) detected significant differences in the presence of bacterial species between vaginal and caesarean deliveries across the 15 studies [29]. Vaginal delivered placentas were enriched with common vaginal microbiota species, such as *Lactobacillus*, *Gardnerella*, *Bifidobacterium*, *Finegoldia*, *Gardnerella*, *Peptoniphilus*, and *Prevotella* [58,60,61,62,63], while placentas derived from cesarean deliveries consisted of bacterial species which typically dominate the skin, such as *Propionibacterium*, *Streptococcus*, and *Staphylococcus* [60,67]. Moreover, placental samples with low microbial signal may be overwhelmed by vaginal microbiota during vaginal delivery, creating difficulty in accurately distinguishing the presence or absence of genuine placental microbes [29]. Therefore, characterization of the placental microbiome should primarily focus on caesarean deliveries. The inconsistent findings across studies raises doubts about the reproducibility and validity of the placental microbiome. Although DNA sequencing is a high throughput technique which allows for the detection and identification of a wide range of bacterial species, it is highly sensitive and is inherently susceptible to the influence of background DNA contamination arising from sample collection and preparation, extraction kits, reagents, and sequencing instruments [68]. This is frequently observed in samples with low microbial biomass such as the placenta, as demonstrated in multiple studies [58,59,60]. Differences in sample size and methodologies used across studies may also contribute to the conflicting results observed. Studies using a standardized approach are needed to fully understand the placental microbiome during pregnancy and its potential impact on maternal and fetal health.

## 4. The Maternal Microbiome during Gestational Diabetes Mellitus

**Figure 3:**
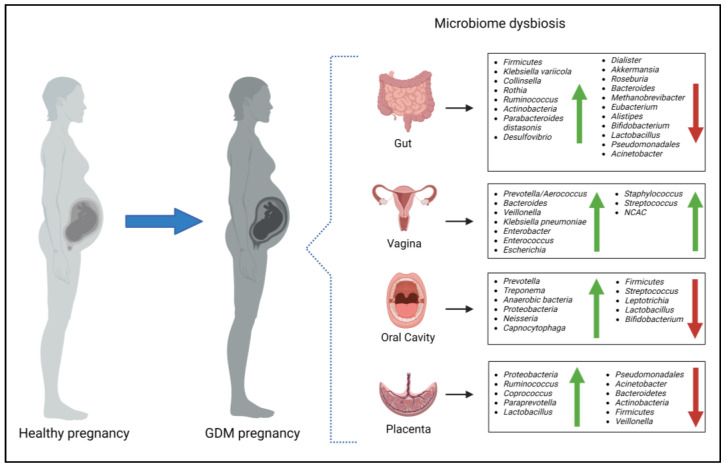
Dysbiosis of the maternal gut, vaginal, oral, and placental microbiome in women with gestational diabetes mellitus (GDM) (Created with BioRender.com). NCAC: non-*Candida albicans Candida* species. Green arrows indicate increased microbiota and red arrows indicate decreased microbiota.

Alterations in the maternal microbiome have been associated with a range of pregnancy complications, including GDM [69,70]. The exact mechanisms by which alterations in the maternal microbiome contribute to the development of GDM are not yet fully understood. However, it is believed that changes in the gut microbiome can lead to increased intestinal permeability, which allows for the translocation of bacterial components into the bloodstream. These bacterial components, such as lipopolysaccharides (LPS), have been shown to activate the innate immune system and promote the release of proinflammatory cytokines. This inflammation may contribute to insulin resistance and impaired glucose tolerance [71]. In addition to alterations in the gut microbiome, changes in the oral microbiome have been associated with the development of GDM. Studies have shown that women with GDM have a higher abundance of pathogenic oral bacteria, which produce inflammatory mediators that can increase insulin resistance and impair glucose tolerance [72]. While data on the vaginal microbiome and GDM is scant, evidence suggest that hyperglycemia during pregnancy is associated with higher rates of vaginal infections [73,74] and that both hyperglycemia and vaginal dysbiosis may lead to poor maternal and neonatal outcomes [11,19]. However, the existence of the placental microbiome is debatable [29]. Research findings have suggested a link between placental microbiome dysbiosis and inflammation, oxidative stress and insulin resistance, which are hallmarks of GDM [66,75,76]. An overview of the microbiota changes that occur in women with GDM are illustrated in Figure 3.

### 4.1. The Gut Microbiome and Gestational Diabetes Mellitus

Evidence suggests that there is a relationship between the gut microbiome and the development and progression of GDM. The microbiome of women with GDM has demonstrated a distinct profile characterized by decreased levels of *Pseudomonadales*, *Dialister*, *Akkermansia*, *Roseburia*, *Bacteroides*, *Methanobrevibacter smithii*, *Eubacterium* species, *Alistipes* species and beneficial bacteria such as *Bifidobacterium* and *Lactobacillus* [77,78], and increased levels of *Firmicutes*, *Klebsiella variicola*, *Collinsella*, *Rothia*, *Ruminococcus*, *Actinobacteria*, *Parabacteroides distasonis*, and *Desulfovibrio* compared to pregnant women with normoglycemia [77,79,80], although with conflicting findings [11]. Variability in the gut microbiome is largely influenced by genetic and individual/environmental characteristics such as age and dietary habits, which may account for differences observed between studies. Moreover, Kuang et al. (2017) demonstrated that GDM-enriched bacteria were positively correlated with glucose levels, providing support for the proposed association with GDM pathophysiology [77]. Recently, studies have shown that the gut microbiome in women with GDM remains dysbiotic until approximately 16 months postpartum [79,81], while another study investigating the gut microbiome five years postpartum found no difference between women with GDM compared to women without GDM [82]. These findings indicate that a prolonged postpartum period might lead to the restoration of the gut microbiome from a state of dysbiosis. Prospective studies are warranted to explore whether microbiota disruption during pregnancy and postpartum is associated with an increased risk of developing T2DM.

### 4.2. The Vaginal Microbiome and Gestational Diabetes Mellitus

Recent studies have highlighted the association between vaginal dysbiosis and hyperglycemia during pregnancy, and the potential role of the vaginal microbiota in the development of GDM [11,18,19,83,84,85]. Cortez et al. (2019) reported an increase in the presence of a rare genera of microbial species, *Bacteroides Veillonella*, *Klebsiella pneumonia*, *Enterobacter*, *Enterococcus* and *Escherichia* in vaginal samples of women with GDM compared to women without GDM [11]. Similarly, Wang et al. (2018) identified differences in the vaginal microbiome composition in women with and without GDM. However, the prevalent genera they identified differed from those observed by Cortez et al. (2019). Instead, the authors reported a correlation between increased ratios of *Prevotella*/*Aerococcus* in the vaginal microbiota and high blood glucose values [85]. Rafat et al. (2022) reported that *Staphylococcus* species were the dominant species in the vaginal microbiome of women with GDM, which was followed by *Streptococcus* species and *Klebsiella* [18]. Notably, *Klebsiella* was previously identified in women with GDM in another study [11]. Furthermore, vaginal infection rates are higher in women with GDM compared to healthy pregnant controls [19]. Accordingly, studies reported an increased prevalence of *non-Candida albicans Candida* species (NCAC) associated with vaginal infections in pregnant women with GDM [18,83]. Moreover, elevated glycemia within vaginal tissue is known to enhance fungal adhesion and proliferation, potentially aiding in the attachment of vaginal epithelial cells to *Candida albicans* cells. Despite variations in the vaginal microbial composition observed across studies, these findings offer a promising avenue for the development of GDM biomarkers, particularly if collected during early pregnancy.

### 4.3. The Oral Microbiome and Gestational Diabetes Mellitus

In recent years, a growing body of evidence indicate that the oral microbiome of women with GDM differs from that of women with healthy pregnancies. Several studies have suggested an association between GDM and periodontitis, a chronic inflammatory condition of the gum tissue, which is caused by pathogenic periodontal bacterial infections [72,86,87]. Higher rates of women with GDM have periodontitis, which is associated with an imbalance in the oral microbiota composition. The oral microbiomes of these women are characterized by increased periodontitis-associated bacteria (*Prevotella*, *Treponema*, and anaerobic bacteria) and a depletion of bacteria associated with the maintenance of periodontal health (*Firmicutes*, *Streptococcus*, and *Leptotrichia*). Wang et al. (2018) reported a positive correlation between glucose levels and the *Neisseria*/*Leptotrichia* ratio in the oral microbiome of pregnant women [85], suggesting a role for these bacteria in the development of GDM. According to a recent review, several studies have similarly showed increased levels of *Proteobacteria*, *Neisseria*, *Prevotella*, and *Capnocytophaga* and reduced levels of *Streptococcus*, *Firmicutes*, and *Leptotricia* [72] in the oral cavity of women with GDM compared to women with healthy pregnancies. Moreover, the abundance of beneficial bacteria such as *Lactobacillus* and *Bifidobacterium*, has been found to be lower in women with GDM compared to women with healthy pregnancies. Differences in the oral microbiota observed between studies may be due to several factors including, genetic differences between study populations, method of bacterial identification, oral health, and sample collection. Further studies using standardized methods and conducted in various populations are required to assess the role of the oral microbiome in women with GDM.

### 4.4. The Placental Microbiome and Gestational Diabetes Mellitus

The presence of a distinct placental microbiota profile has been associated with GDM and metabolic dysregulation [66,75,76]. Specifically, women with GDM exhibited reduced levels of *Pseudomonadales* and *Acinetobacter* compared to women with healthy pregnancies. Moreover, the decreased abundance of placental *Acinetobacter* was associated with more adverse metabolic and inflammatory phenotypes, and lower placental expression of several anti-inflammatory genes such as interleukin (*IL-10*) [75]. In another study, four dominant phyla, *Bacteroidetes*, *Firmicutes*, *Actinobacteria*, and *Proteobacteria*, were identified in the placenta [76]. Of these, *Proteobacteria* was shown to be increased and *Bacteroidetes*, *Actinobacteria* and *Firmicutes* were decreased in women with GDM compared to healthy pregnant women. The levels of these phyla were found to correlate with various clinical characteristics of both the mother and the infant, such as cord blood insulin, insulin-like growth factor-1 (*IGF-1*), and leptin levels. These associations suggest that the placental microbiota may potentially play a role in regulating glucose metabolism, fetal development and growth [76]. However, these studies are cross-sectional, and no definitive causal relationship can be established. Aside from the prominent phyla observed in the placental microbiome, Tang et al. (2020) found higher levels of the *Ruminococcus*, *Coprococcus*, *Paraprevotella*, and *Lactobacillus* genus, and reduced levels of *Veillonella* in the placentas of women with GDM compared to healthy pregnant women [66]. The variations observed in the placental microbiome in GDM studies may be due to multiple factors, including the potential risk of contamination during mode of delivery and sample collection, as previously discussed. To better understand the link between the placental microbiome and GDM, additional research and evidence are necessary to investigate the association and underlying mechanisms.

## 5. The Microbiome in Pregnancy Complications

Women with GDM have an increased risk of developing obstetrical complications such as preterm labor and birth, gestational hypertension and preeclampsia, and excessive weight gain [17,27], which may in turn have a profound impact on the health of the neonate and infant. Whilst the etiology of these pregnancy complications is not completely understood, a microbial role has been implicated. The current available data regarding the maternal microbiome and its association with maternal complications are discussed below.

### 5.1. Preterm Birth

Preterm birth, defined as birth that occurs before 37 weeks of gestation, is a major cause of neonatal morbidity and mortality worldwide [88]. Increasing evidence link the vaginal and oral microbiomes to the risk of spontaneous preterm labor [17,89]. Pregnant women with low levels of *Lactobacillus crispatus* and a wider variety of bacterial species in the vaginal microbiome are at higher risk for preterm delivery compared to women with high levels of *Lactobacillus crispatus* [89]. In addition, bacterial vaginosis, which is characterized by an overgrowth of harmful bacteria such as *Prevotella bivia*, *Peptostreptococcus*, and/or *Garnerella vaginalis* in the vagina, has been associated with an increased likelihood of preterm labor and delivery [90,91]. Moreover, an increased susceptibility to preterm birth was associated with the presence of vaginal fungi such as *Candida albicans* [92]. These findings suggest that the diversity of the vaginal microbiome may play a role in the risk of preterm birth, were women with decreased levels of protective bacterial species and increased levels of harmful bacteria may be at greater risk. In contrast to these findings, a study conducted in an African population showed no association between preterm birth and the composition of the vaginal community [93]. Differences observed between these studies may be due to sample size and ethnicity. The oral microbiome has also been correlated with preterm birth. Studies assessing the relationship between the oral microbiome and pregnancy complications, have shown that hormonal changes during pregnancy may promote the formation of bacterial plaques, thereby resulting in gingivitis and/or periodontitis [27,94]. Accordingly, a review of the literature and meta-analysis including 12,407 pregnant women showed a correlation between gingivitis and periodontitis and increased risk of preterm birth [27,94]. These findings emphasize the need for dental hygiene as an integral component of prenatal care. Recently, the placental microbiome has been associated with preterm birth [95,96], although conflicting findings exist [93,97]. Discrepancies observed between studies may be due to difficulty in recovering viable DNA samples from the placenta or the detection of low bacterial loads that are indistinguishable from negative controls or potential sources of contaminants. Understanding the variables linked to changes in the microbiota composition may have important implications for reducing the risk of preterm birth and enhancing reproductive health outcomes.

### 5.2. Gestational Hypertension and Preeclampsia

The complex interplay between gestational hypertension, preeclampsia, and GDM is an area of ongoing research, and at this point, it is more appropriate to consider it as a correlation rather than a definitive causation. Studies have observed that these conditions often occur together in pregnant individuals more frequently than what would be expected by chance alone. This correlation suggests that there may be shared risk factors such as maternal age, obesity, family history, and race/ethnicity, or biological mechanisms at play that contribute to their concurrent occurrence. Additionally, insulin resistance, oxidative stress and inflammation are known to play a role in the development of both GDM and preeclampsia [98]. These overlapping risk factors suggest that there may be common pathways influencing the development of these conditions [99].

The placenta plays a crucial role in pregnancy, and dysfunction in this organ has been implicated in both gestational hypertension and preeclampsia [98,100]. The exact mechanism linking GDM to hypertensive diseases of pregnancy are complex and not completely understood, but there are several factors at play [99]. The pathophysiological process of preeclampsia involves two stages. In the first stage, inadequate invasion of trophoblasts during early pregnancy leads to incomplete remodeling of spiral arteries, resulting in reduced blood supply to the placenta and the development of placental ischemia and oxidative stress. The placenta in this state releases higher levels of anti-angiogenic factors such as soluble fms-like tyrosine kinase-1 (sFlt1) and soluble endoglin. These factors contribute to inflammation and dysfunction of maternal blood vessels, ultimately leading to systemic health issues. In the context of GDM, hyperglycemia plays a role in aggravating the pathophysiological process. Hyperglycemia induces inflammation and autophagy in trophoblasts, which impairs their ability to migrate and invade maternal tissues. In GDM, neutrophils become overactive and release excessive neutrophil extracellular traps (NETs). These NETs obstruct blood circulation within the villous space of the placenta, and contributes to placental ischemia, which is closely linked to the onset of preeclampsia. Oxidative stress is heightened in both GDM and preeclampsia. Hyperglycemia triggers oxidative stress through various mechanisms, including the formation of advanced glycation end products. This leads to increased production of reactive oxygen species, which in turn reduces the availability of nitric oxide, a molecule important for blood vessel dilation, and consequently leads to impaired vasodilation. Inflammatory markers such as tumor necrosis factor-α (*TNF-α*) and Interleukin-6 (*IL-6*) are elevated in GDM and preeclampsia. These cytokines are associated with endothelial dysfunction and are considered independent risk factors for preeclampsia in women with GDM [99]. Moreover, insulin resistance, a key feature of GDM, has also been linked to endothelial dysfunction, which plays a role in the development of preeclampsia. It is hypothesized that insulin resistance may contribute to the inflammatory response and impaired vascular function seen in preeclampsia [101].

Although little is known about the impact of the microbiome, recent studies have linked the maternal gut, placenta, and oral microbiome to the development of gestational hypertension and preeclampsia.

Significant changes in the gut microbiota of women with preeclampsia have been demonstrated. A study conducted in Chinese women with preeclampsia showed that the gut microbiota profile in the third trimester of pregnancy differed significantly compared to women with healthy pregnancies [102], and was characterized by an increased abundance of pathogenic bacteria, *Clostridium perfringens* and *Bulleidia moorei*, and a decrease in probiotic bacteria *Coprococcus catus*. Furthermore, a study conducted by Chen et al. (2020) demonstrated an increased abundance of opportunistic pathogens such as *Fusobacterium* and *Veillonella*, while beneficial bacteria such as *Faecalibacterium* and *Akkermansia* were markedly depleted in the gut of women with preeclampsia during the third trimester [103]. Early onset preeclampsia (diagnosed before 34 weeks) tends to be more serious than late-onset preeclampsia and is thought to have a different etiology [104]. During early pregnancy, Gomez-Arango et al. (2016) showed a negative correlation between the abundance of *Odoribacter*, a butyric acid-producing bacterium, and systolic blood pressure, a hallmark of gestational hypertension and preeclampsia [105]. Additionally, Lv et al. (2019) identified an association between changes in the gut microbiota and early-onset preeclampsia, which was correlated with maternal clinical features such as blood pressure and liver dysfunction [106]. The authors reported that eight bacterial genera were significantly enriched in the microbiomes of women with preeclampsia compared to healthy controls, of which *Blautia*, *Ruminococcus,* and *Bilophila* were reported to have the major variances between women with preeclampsia and normal pregnancies [106]. Moreover, gut microbiota such as *Bilophila*, *Oribacterium*, and *Akkermansia*, were correlated with host immune parameters such as *IL-6*, and LPS, a major bacterial component which can trigger a significant immune response. These findings suggest that an altered gut microbiome during early pregnancy may be involved in the development of early-onset preeclampsia by acting on the maternal immune system and the production of proinflammatory cytokines. Gastrointestinal, respiratory, and oral infections have been implicated in the pathogenesis of preeclampsia [27,107,108]. In a recent study, various bacteria found in the placenta have been associated with these infections. For example, *Escherichia*, *Bacillus*, *Salmonella*, and *Listeria* have been linked to gastrointestinal infections, while *Anoxybacillus* and *Klebsiella* are associated with respiratory infections. Additionally, *Dialiste*, *Variovorax*, *Porphyromonas*, and *Prevotella shahii* have been associated with periodontitis [108]. Moreover, oral bacteria associated with gingivitis or periodontitis have been linked to an increased risk of preeclampsia [27,107], but should be interpreted with caution due to their multifactorial etiologies.

The precise mechanisms underlying the correlation between gestational hypertension, preeclampsia, and GDM are not fully understood and may vary between individuals. While there is a clear correlation between these conditions, it is challenging to establish causation definitively due to the complex nature of pregnancy-related complications and the potential involvement of multiple factors. The above evidence suggests that the gut microbiome plays a vital role in early and late onset preeclampsia in pregnant women. In addition, findings from these studies indicate that the imbalance of placental and oral microbiomes may be closely related to the occurrence and development of preeclampsia. Further research is needed to better understand the causal relationships, if any, between these conditions. As more evidence emerges, we may gain a clearer understanding of whether there are causal links between gestational hypertension, preeclampsia, and GDM or if their co-occurrence is primarily due to shared risk factors and biological pathways.

### 5.3. Gestational Weight Gain

Excessive weight gain during pregnancy is associated with several adverse pregnancy outcomes [109]. Increasing evidence suggests that changes in metabolic, hormonal, gastrointestinal permeability and associated metabolic disturbances that occur during pregnancy may impact the composition of the gut microbiota and consequently, maternal weight gain [110]. Accordingly, studies have shown that excessive weight gain during pregnancy is correlated with gut microbiome dysbiosis [78,111]. A prospective follow-up study showed that excessive weight gain during pregnancy was associated with high levels of *Bacteroides* species and *Staphylococcus* in the gut microbiome [78]. Similarly, Santacruz et al. (2010) reported an increase in *Staphylococcus*, *Enterobacteriaceae*, and *Escherichia coli* in the gut of overweight pregnant women compared to normal-weight pregnant women, while the abundance of *Bacteroides* and *Bifidobacterium* were decreased [111]. The study further showed that the increased abundance of bacterial species was associated with weight gain and biochemical parameters such as transferrin, plasma cholesterol, high-density lipoprotein cholesterol, folic acid, and triglyceride concentrations. Taken together, these studies indicate that the gut microbiome is closely related to excessive weight gain and gestational obesity during pregnancy and may play an important role in regulating maternal lipid metabolism. The mechanism by which the gut microbiome influences gestational weight gain is not clear, although several mechanisms involving nutrient metabolism, hormone regulation, inflammation and immune response, short chain fatty acid (SCFA) production, and the gut–brain axis have been proposed [78,112,113,114]. Regulating the gut microbiome to improve excessive weight gain during pregnancy may be a promising and novel strategy.

## 6. Alterations in the Microbiome and Neonatal Complications

The maternal microbiota is not only closely related to the health of the mother but may also affect the health of the offspring. The newborn microbiota is strongly influenced by the intrauterine environment, and when dysregulated, may have an impact on developmental programming and long-term neonatal outcomes [17,85].

Evidence suggest that the development and composition of the infant microbiome is related to maternal sources of microbial transmission [115]. In a longitudinal study, Ferreti et al. (2018) assessed the early acquisition and development of the infant gut and oral microbiome, and the role of the maternal microbiome. Samples from 25 mother–infant dyads were collected at multiple sites (skin, breast milk, stool, vagina, and oral cavity) from mothers and from the stool and oral cavity of infants at birth to four months postpartum [115]. The authors reported that the maternal gut microbiome was the major source of transmission for infant-acquired strains in the gut microbiome, while the oral cavity appeared to be the least important route of transmission. Furthermore, prevalent strains within the infant gut microbiome were also detected in the maternal skin and vaginal microbiomes, albeit to a lesser extent. In addition, the authors reported a dramatic decrease in the microbial diversity and strain heterogeneity within the first week after birth, before recovering and gradually increasing over the next four months. These findings support the hypothesis of vertical mother to child transmission, which is integral to the development of the infant microbiome within the first four months after birth [115]. Accordingly, studies have shown that childbirth and postpartum are the most significant periods for vertical transmission, especially when infants are exposed to maternal skin, breast milk, vagina, and feces [116,117,118,119].

At birth, the infant continues to acquire microorganisms. Various factors have been associated with the initial development of the infant gut microbiome composition, including mode of delivery, feeding, gestational age at birth and antibiotic usage [115]. Findings from a 7-year longitudinal mother–child-dyad study showed that children born to mothers who had taken antibiotics late in the third trimester had an 84% risk of developing childhood obesity, while children born via caesarean section had a 46% risk [120]. Caesarean deliveries may lead to exposure of the neonate to maternal skin and environmental microorganisms, whereas vaginal delivery exposes the neonate to the maternal vaginal microbiota. Additionally, antibiotic administration during early infancy has been linked to a higher risk of early childhood obesity [121]. These findings suggest that early exposure of the infant to the maternal microbiome alters the composition of the infant’s gut microbiome and may predict excessive weight gain in offspring during childhood and later in life.

After birth, breastfeeding continues to expose the newborn’s gut to new maternal microbiota. Thus, the relationship between the breast milk microbiome and the infant gut microbiota in the postpartum period has attracted increasing attention. A review conducted by Milani et al. (2017) reported that the gut microbiome of breastfed infants exhibits an increased abundance of healthy bacteria, *Bifobacteria* and *Lactobacilli*, and reduced levels of potential pathogens compared to formula-fed infants [122]. Furthermore, *Bifobacteria* and *Lactobacilli* species are known to activate immunoglobulin A-producing plasma cells in the neonatal gut and has been associated with immune system regulation [123]. A prospective study demonstrated that the bacterial species present in the mother’s breast milk are the most prevalent within the gut microbiota of infants in the first month postpartum [124], thus illustrating a link between breastfeeding and the early-life gut microbiota of the infant.

Studies investigating a possible causal link for disease programming suggest that dysbiosis of the gut microbiome negatively affects offspring metabolic health [17]. The gut microbiota of full-term infants consists primarily of anaerobic organisms immediately after birth, and is initially colonized by facultative anaerobic organisms, followed by obligate anaerobes, including *Bifidobacterium*, *Bacteroides*, and *Clostridium*. These anaerobes are associated with immune modulation, host–gut crosstalk, and mediating microbiota colonization [125]. However, disruption of the infant gut microbiome during early life has been associated with pediatric disorders, and the onset of inflammatory, immune-mediated, allergic, and cardiometabolic diseases in later life [126,127]. Although the microbial alterations that lead to gut dysbiosis is not completely understood, environmental and physiological triggers may in part induce these changes. Moreover, microbiota-related epigenetic changes during early development may also affect phenotypic characteristics associated with metabolic disease later in life [125].

Recent research reported that GDM alters the newborn’s gut microbiome, which contributes to the current understanding of intergenerational obesity and diabetes prevalence [128]. Offspring born to mothers with GDM exhibit reduced microbial diversity of various bacterial types compared to offspring of healthy mothers, indicating possible gut dysbiosis in the offspring. Moreover, offspring exposed to GDM may be predisposed to develop gastrointestinal disease, childhood obesity and metabolic syndrome in later life, thereby affecting future health [129,130].

Overall, the maternal microbiome can have a significant impact on the development of the neonatal microbiome during pregnancy and postpartum. Understanding these relationships may aid in the development of interventions that promote the growth of healthy microbiota in infants, which may have long-term implications for their health and development.

## 7. Limitations

Maternal microbiome studies provide valuable insights into the relationship between the mother’s microbial composition and various aspects of pregnancy outcomes and maternal and infant health. However, the variation in the maternal microbiome across studies included in this review, highlight key challenges that must be addressed.

Technical limitations that may influence the discrepancies observed between microbiome studies includes, methods of detection, bioinformatic pipelines, lack of standardization and sample storage conditions. The most common techniques for detecting microbial composition include, 16S rRNA marker gene sequencing and shotgun metagenomic sequencing [19,63,65,85], quantitative real-time PCR (qRT-PCR) of the 16S rRNA gene or specific probes for selected taxa [59] and cell culture experiments [18,60,83,131], with studies showing diverse findings when comparing microbial species using two or more techniques [33,60]. The lack of consistency in the analytical pipeline used for processing DNA sequence data, including decisions regarding how sequences should be grouped or split into taxonomic units, may also contribute to the variability observed. Moreover, qRT-PCR provides an affordable and easy method to monitor changes in the microbiota; however, it is limited by its ability to only identify known, dominant microbial taxa [132]. In the current review, several studies lacked positive controls for standardization, leading to challenges in accurately differentiating between the presence of bacterial species and potential background contamination in the samples [63,64,65,66]. Alternative techniques, such as bacterial cultures, enable a targeted analysis of specific bacterial species or strains within the microbiome [18,60,83,131]. This method presents an opportunity to explore the functional properties of microbial species and understand how microbial factors influence host cells. However, cell culture studies are limited by their ability to only identify putative anaerobic species, which may potentially exclude important microbial species from the analysis, leading to bias in the results reported. Other factors such as freezing of samples and growth and preservation media may also influence recovery of bacteria in cell culture experiments [60,61].

Differences between studies may be due to sample size, study design, gestational age at sample collection, method of delivery, and GDM treatment. A large sample size is commonly favored to enhance statistical power. However, recruiting an adequate number of women, particularly those with specific conditions such as GDM, can be challenging. Apart from a few studies that have sample sizes exceeding 500 [58,65,85], the majority of studies included in this review have relatively small sample sizes, which limits the generalizability of the findings [11,59,62,66,82,83,133]. Several studies suggest that the maternal microbiome changes during pregnancy, between gestation and postpartum and in relation to several pregnancy associated metabolic factors [33,79,82]. Therefore, a single timepoint sampling, as in the majority of studies included in this review, may not fully capture the complexity of microbiome changes at different stages during and after pregnancies. Collecting data at multiple time points is essential to gain deeper insight into the causal link between the maternal microbiome, GDM, and pregnancy outcomes. In addition, timing and method of delivery significantly influences microbial composition, particularly when assessing the placental microbiome. For example, studies investigating the placental microbiome in samples delivered vaginally compared to caesarean deliveries showed large variations in microbial composition, possibly due to contamination from delivery pathways [29]. It is well known that energy intake influences microbial composition [37,134]. Thus, GDM treatment by diet compared to antidiabetic medication may account for microbial differences observed across studies [11,18,85].

Several studies have reported substantial variation in microbial populations due to interindividual heterogeneity [11,63,77,79]. Confounding factors such as genetics, maternal age, ethnicity, dietary intake, body mass index (BMI), antibiotic usage, and medical and environmental conditions, which are not always accounted for during analysis, influence the microbial composition of pregnant women and may account for the inconsistencies reported in the findings of this review. This highlights the importance of replicating findings in larger, more diverse populations to validate the observed associations.

Addressing drawbacks and limitations is crucial to ensure the reliability, validity, and clinical utility of the microbiome in health care and research settings. Continued advancements in study design, technology, and data analysis techniques are essential for overcoming these challenges and harnessing the full potential of microbiome studies.

## 8. Future Perspectives and Therapeutic Potential

The maternal microbiome holds great promise for improving maternal and infant health. Identifying modifiable factors that restore dysbiosis of the maternal and infant microbiome may support health strategies to reduce pregnancy complications such as GDM and related adverse outcomes.

As a strategy to reduce the risk of GDM and related adverse pregnancy outcomes, the attainment of a healthy maternal microbiome before and during pregnancy through the promotion of a balanced diet, normal weight gain during pregnancy, and oral hygiene should be targeted. Various micronutrients such as vitamins, minerals, and antioxidants obtained through diet can influence the maternal gut microbiome. For example, vitamin D has been associated with a more diverse gut microbiota and plays a relevant role in maintaining immune system function [135]. Moreover, certain foods such as yogurt and fermented vegetables contain probiotic strains, which introduce beneficial bacteria into the maternal gut, and can potentially modulate the immune response, reduce inflammation and enhance gut barrier function [136,137,138]. In addition, diets high in fiber promote the growth of beneficial bacteria that ferment dietary fiber into SCFAs, providing energy for the intestinal cells [139]. On the other hand, diets high in simple sugars and saturated fats can favor the growth of less desirable microbial species associated with inflammation and metabolic disorders [140]. Moreover, high glycemia compromises the integrity of the gut barrier, which allows the translocation of bacterial components into the bloodstream, a phenomenon known as leaky gut or increased intestinal permeability [141]. The translocation of bacterial components can trigger an inflammatory response and can contribute to the development of chronic low-grade inflammation, which further disrupts the gut microbiome, as seen in diabetes [141]. This suggests that there is a clear bidirectional relationship between glycemia and the microbiome. Thus, restoring the gut microbial composition through dietary modifications, lifestyle changes and targeted interventions may help improve glycemic control, and in turn provide an opportunity to develop targeted strategies for modulating the microbiome in the context of GDM management. Consulting a healthcare professional or registered dietitian who specializes in prenatal nutrition can provide personalized dietary recommendations to optimize the maternal microbiome before and during pregnancy.

Many pregnant women who are exposed to antibiotics during pregnancy or at delivery may experience a significant shift in the gut microbiome, which results in the suppression of both beneficial and pathogenic bacteria. This could potentially alter the first fetal–microbial interactions, which influences the development of the infant microbiome. Administration of probiotics may help to restore or improve the dysbiosis of the maternal microbiome during pregnancy or labor. Probiotics are microorganisms that promote health benefits to the host, with *Bifidobacterium* and *Lactobacillus* being the most widely used [142]. The use of probiotics can regulate and promote a healthier composition of the intestinal microbiota, inhibit pathogen colonization, strengthen intestinal permeability, and modulate the immune response, insulin signaling, and energy metabolism [143]. During pregnancy, probiotics have been proven to exert beneficial effects in various clinical conditions, including GDM [144,145], and may offer a safe alternative for the prevention of GDM. In a recent systematic review and meta-analysis of data from 14 randomized control trials, Yefet et al. (2023) reported that probiotic administration in women with GDM were associated with a reduction in the levels of plasma fasting glucose, insulin, triglycerides, total cholesterol, and very low-density lipoprotein (VLDL) compared to pregnant women who received the placebo [146]. The authors reported that *Lactobacillus acidophilus*, *Bifidobacterium bifidum*, and *Lactobacillus casei*, were the most common bacterial strains used in the probiotic formulas across studies, which had favorable effects on metabolic outcomes when assessed separately. Interestingly, a decrease in neonatal birth weight was observed in women receiving probiotic supplements containing the *Lactobacillus acidophilus* bacterial strain compared to the control group. These findings suggest that probiotic supplementation during pregnancy may improve glycemic control and lipid profiles and reduce the risk of unfavorable maternal and fetal outcomes in women with GDM. However, future studies should examine the effect of each bacterial strain, to characterize the appropriate probiotic supplement needed to treat a specific metabolic disorder [146].

The key targets for promoting a healthy infant microbiome include the promotion of breastfeeding and kangaroo care in the perinatal period [17]. Breast milk is a source of essential nutrients and bioactive compounds, including beneficial bacteria which shapes the colonization of the infant gut [147]. Thus, regular and exclusive breastfeeding promotes the transmission of beneficial bacteria to the infant, positively influencing their gut microbiota. Moreover, the physical contact involved in kangaroo care facilitates the transmission of maternal skin microbiota to the infant skin microbiome. This exposes the infant to a wide range of microbial species that play a crucial role in immune system development [148]. In addition, kangaroo care has also been associated with improved gut maturation in premature infants with underdeveloped digestive systems [149]. Thus, investigating the interplay between the maternal microbiome, breast milk composition, and infant health could lead to targeted interventions to promote healthy microbial colonization.

Findings from this review suggest that the development of strategies to modulate the gut microbiota may significantly impact maternal and fetal health and their future risk for metabolic diseases. Furthermore, personalized medicine approaches that consider an individual’s genetic and environmental factors may help tailor interventions to specific needs, thereby optimizing outcomes.

## 9. Conclusions

There is a need for future research to elucidate maternal microbiome patterns that protect against the risk of adverse pregnancy outcomes that impact maternal, neonatal, and infant health. Continued research and clinical trials are necessary to fully realize the therapeutic potential of the maternal microbiome and translate these findings into clinical practice.

## Data Availability

No new data were created.
